# An Evaluation of the Accuracy of Classical Models for Computing the Membrane Potential and Extracellular Potential for Neurons

**DOI:** 10.3389/fncom.2017.00027

**Published:** 2017-04-24

**Authors:** Aslak Tveito, Karoline H. Jæger, Glenn T. Lines, Łukasz Paszkowski, Joakim Sundnes, Andrew G. Edwards, Tuomo Māki-Marttunen, Geir Halnes, Gaute T. Einevoll

**Affiliations:** ^1^Simula Research Laboratory, Center for Biomedical ComputingOslo, Norway; ^2^Department of Informatics, University of OsloOslo, Norway; ^3^RadytekWrocław, Poland; ^4^Department of Biosciences, University of OsloOslo, Norway; ^5^NORMENT, K.G. Jebsen Center for Psychosis Research, Institute of Clinical Medicine, University of OsloOslo, Norway; ^6^Department of Mathematical Sciences and Technology, Norwegian University of Life SciencesÅs, Norway; ^7^Department of Physics, University of OsloOslo, Norway

**Keywords:** cable equation, membrane potentials, numerical modeling, ephaptic coupling, extracellular potential

## Abstract

Two mathematical models are part of the foundation of Computational neurophysiology; (a) the Cable equation is used to compute the membrane potential of neurons, and, (b) volume-conductor theory describes the extracellular potential around neurons. In the standard procedure for computing extracellular potentials, the transmembrane currents are computed by means of (a) and the extracellular potentials are computed using an explicit sum over analytical point-current source solutions as prescribed by volume conductor theory. Both models are extremely useful as they allow huge simplifications of the computational efforts involved in computing extracellular potentials. However, there are more accurate, though computationally very expensive, models available where the potentials inside and outside the neurons are computed simultaneously in a self-consistent scheme. In the present work we explore the accuracy of the classical models (a) and (b) by comparing them to these more accurate schemes. The main assumption of (a) is that the ephaptic current can be ignored in the derivation of the Cable equation. We find, however, for our examples with stylized neurons, that the ephaptic current is comparable in magnitude to other currents involved in the computations, suggesting that it may be significant—at least in parts of the simulation. The magnitude of the error introduced in the membrane potential is several millivolts, and this error also translates into errors in the predicted extracellular potentials. While the error becomes negligible if we assume the extracellular conductivity to be very large, this assumption is, unfortunately, not easy to justify *a priori* for all situations of interest.

## 1. Introduction

Computational modeling in neurophysiology is a rapidly developing field taking on problems of enormous complexity. This is illustrated in the recent paper by Markram et al. (2015) where the authors present results of amazingly detailed digital algorithmic reconstruction of a neocortical volume segment (about 0.29 mm^3^) of rat cortex, containing ~31,000 neurons with ~37 million synapses. The complexity of the project is astonishing, and it opens amazing perspectives for insight in the complexities of the brain. The paper also raises questions of more philosophical nature brilliantly examined in the accompanying perspective by Koch and Buice (2015).

The development of enormously complex computational models motivates closer examination of the basis of the mathematical models underpinning the computations. It is the purpose of this study to evaluate the accuracy of two basic models extensively used throughout the field of computational neurophysiology, and our main question is whether the popularity of these models is warranted by their accuracy.

The first model we consider is the celebrated Cable equation used to compute membrane potentials and transmembrane currents. This model is absolutely essential in computational neurophysiology, and is used in numerous papers every year. The derivation of the model is classical and can be found in any introduction to computational neurophysiology; see e.g., Sterratt et al. (2011), Ermentrout and Terman (2010), Scott (2002), Dayan and Abbott (2001), and Koch (1999). An important assumption in the most common derivation of the Cable equation is that the extracellular conductivity is very large, and that consequently the extracellular potential can be assumed to be constant. This assumption represents a major simplification of the model since the extracellular field does not have to be represented in the model, which means that a costly solution of a Poisson equation in the extracellular domain is avoided.

One way of interpreting the effect of ignoring the coupling to the extracellular potential is that (as we shall see below) we disregard the so-called ephaptic current; see e.g., Holt and Koch (1999). It is well known that neglecting this current represents a key assumption, and the validity of the assumption, and also the effect of ephaptic coupling, have previously been discussed by several authors; see e.g., Buzsáki et al. (2012), Bhalla (2012), Goldwyn and Rinzel (2016), Anastassiou et al. (2011), Anastassiou and Koch (2015), and Bokil et al. (2001). An analytical treatment of the effect of ephaptic currents on nerve pulses in parallel nerve fibers is given in Chapter 8 of Scott (2002). That exposition is motived by classical experiments performed by Katz and Schmitt (see e.g., Katz and Schmitt, 1940) and analyzed by an extension of the scalar Cable equation to a 2 × 2 system of partial differential equations governing the membrane potential of the neighboring nerve fibers. This work is followed up by Shneider and Pekker (2015) who suggest that the ephaptic current acts as a synchronization mechanism for action potentials along bundles of neurons. For axons, the coupling is particularly important in the unmyelinated case; see Bokil et al. (2001) for an analysis of bundles of olfactory nerve axons. Furthermore, Goldwyn and Rinzel (2016) recently studied ephaptic interactions in a bundle of neurons and found that the effects of the ephaptic currents were small but not negligible.

The question of ephaptic coupling between cells has been studied for a long time; 75 years ago (Arvanitaki, 1942) stated that *there is no doubt that the activity of an element in the midst of a cell agglomeration can influence that of its neighbors, even when specialized contact surfaces for transmission, i.e., those loci traditionally known as synapses and which have been endowed with particular properties are lacking*. In Holt and Koch (1999), Holt and Koch analyse the magnitude and possible consequences of ephaptic coupling. They observe that spikes from a neuron can cause an extracellular potential of a few mV near the cell body, and they analyse the effect of this on nearby cells. The impact of ephaptic coupling remains uncertain (Anastassiou et al., 2011; Anastassiou and Koch, 2015), but it seems to be acknowledged that ephaptic currents *may* be significant. However, it is usually not taken into account in most computational analyses of neurons, and the reason for this is clearly to improve computational efficiency. In this paper we will quantify the error introduced by this assumption. We will compare the results of the Cable equation to those of an accurate mathematical model which includes the ephaptic current. The more accurate model will be referred to as the **EMI** model since it builds on detailed representation of both the **E**xtracellular space surrounding the neuron, the **M**embrane of the neuron and the **I**ntracellular space of the neuron. EMI computations are typically *much* more CPU demanding than solving the Cable equation, but the model faithfully represents the physics of the neuron and its surroundings. Variants of the EMI model have been studied previously by e.g., Krassowska and Neu (1994), Ying and Henriquez (2007), Henríquez et al. (2013), Agudelo-Toro and Neef (2013), and Agudelo-Toro (2012). For linear membrane currents and specialized geometries, analytical solutions are available; see e.g., Rall (1962), Rall (1969), Klee and Rall (1977), Krassowska and Neu (1994), Ying and Henriquez (2007), and Agudelo-Toro and Neef (2013).

The second model we consider is the standard formalism for computing the extracellular potential based on solutions of the Cable equation. It is well known that if the current sources are given by Dirac delta functions, the solution of the Poisson equation, defined on an infinite domain, can be computed by an explicit formula, see e.g., Einevoll et al. (2013). Based on the solution of the Cable equation, the current sources can be defined for each compartment in the numerical solution, and the solution of the Poisson equation can (due to linearity) be given as the sum of contributions from all compartments. Note that in practice the so-called *line-source approximation* (Holt and Koch, 1999) where the current sources are assumed to be evenly distributed along cylindrical axes of dendritic compartments, is commonly used rather than the point-source approximation built on solutions of the Dirac delta functions. However, these two methods are directly related as a line source can be arbitrarily accurately approximated by a line of delta-function sources.

The combined use of the two models (a) and (b) in computing extracellular potentials is especially intriguing since (a) is solved based on the assumption that the extracellular field is *constant*, and then (b) is used to compute the *non-constant* extracellular potential.

We have evaluated the accuracy of these two basic models by comparing the results with the results obtained by solving the EMI model. Our findings can be summarized as follows:

1. We find that the membrane potential computed by the Cable equation qualitatively resembles the solution of the EMI model but may differ quantitatively (several millivolts) from the solution of the EMI model.
2. We find, using reasonable parameters, that the magnitude of the ephaptic current is comparable to the other currents in our example model, so that its omission is, in general, difficult to justify.
3. For our example application the error in neglecting the ephaptic effect when computing the extracellular potentials is found to be 10% or more, and stem from the inaccurate computation of the transmembrane currents when the extracellular potentials are assumed to be constant.

We have found the EMI model to be a useful framework for assessing the accuracy of the classical models. The EMI model is, however, *much more computationally demanding*, it is much more difficult to implement correctly, and therefore very challenging to apply to problems of greater complexity than the simple problems addressed in the present report.

The rest of this report is organized as follows: In the Methods Section, we derive the classical Cable equation and highlight what assumptions are needed to remove the extracellular potential from the model. Given the solution of the Cable equation, we show how to compute the extracellular potential by solving a boundary value problem, how to approximate the solution by solving a Poisson equation, and how to approximate the solution of the extracellular potential using a classical summation formula. Finally, we introduce the EMI model where the dynamics of the extracellular space, the cell membrane and the intracellular space are fully coupled, and we show how the EMI model can be solved numerically. In the Results Section, we study the error introduced in the model by ignoring the ephaptic currents and how the ephaptic current depends on the extracellular conductivity. Furthermore, we compare the extracellular potential around a single simplified neuron computed by various approximate models, and we also compare the extracellular potential between two simplified neurons. Finally, we show that the numerical solutions seem to converge under mesh refinement and that infinite domains can be reasonably well represented using large extracellular domains. Implications and relevance of the results are examined in the Discussion Section. In an Appendix in Supplementary Material we give a theoretical estimate of the error introduced by removing the ephaptic current.

## 2. Methods

The standard way of computing the extracellular potential surrounding a neuron is a two-step process: (a) solve the Cable equation, and (b) use the transmembrane currents defined by step (a) to compute the extracellular potential. Our aim is to assess the accuracy of the solution of these two steps. For comparison we will use an accurate model combining the Extracellular domain, the Membrane, and the Intracellular domain, referred to as the EMI model. Below, the EMI solution will be regarded as the reference solution, and therefore solutions computed by all other methods (derived below) will be compared to the EMI solution.

We will take care to try to explain exactly how the EMI model and the two-step models are defined and solved although the derivations presented here can, at least in part, be found elsewhere. The derivations will also help us clarify what assumptions underlie the various models.

### 2.1. The classical two-step method

We start by describing the two steps of the classical approach of computing the extracellular potential (Holt and Koch, 1999; Lindén et al., 2014). The first step is to compute the membrane potential and transmembrane currents. In the classical approach this problem is solved assuming a constant extracellular potential. We briefly review the derivation of the Cable equation in order to clarify exactly what assumption is made in order to remove the extracellular potential from the equation defining the membrane potential. By identifying what term is ignored in the equation, this term can be evaluated and used to illuminate the accuracy of the Cable equation.

The second step is to compute the extracellular potential by using the solution of the Cable equation to define the transmembrane current sources. This step can be done in numerous ways, and we will derive alternative methods starting with the approach considered to most faithfully represent the physics involved, and then derive simpler and more efficient methods in order to end up with the classical summation formula defining the extracellular potential.

#### 2.1.1. The cable equation

**Figure 1:**
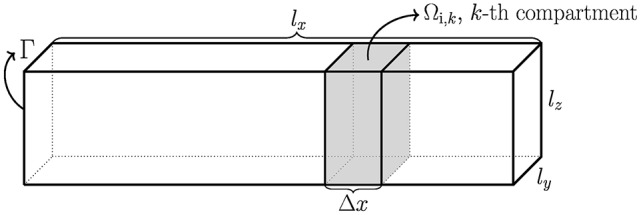
**Sketch of a simplified neuron of rectangular cuboid shape with dimensions *l*_*x*_, *l*_*y*_, and *l*_*z*_**. The intracellular domain is denoted Ω_i_, the boundary is Γ, and the compartments of length Δ*x* are denoted by Ω_i, *k*_.

Consider a simplified neuron geometry illustrated in Figure 1. The intracellular space of the neuron is denoted by Ω_i_ and the boundary of Ω_i_ is the membrane of the neuron, denoted by Γ. The size of Ω_i_ is given by *l*_*x*_, *l*_*y*_, and *l*_*z*_. In the derivation of the Cable equation, the neuron is divided into compartments, see e.g., Sterratt et al. (2011), Scott (2002), and Ermentrout and Terman (2010), and it is assumed that the variations in the *y*- and *z*-directions are small and can be ignored. Our derivation is based on the version of the Cable equation used in Holt and Koch (1999). The compartments are denoted by Ω_i,*k*_, and have length Δ*x*. For the *k*-th compartment, the transmembrane current density (positive outward) is given by (see e.g., Sterratt et al., 2011)

(1) $$\begin{array}{r} I_{\text{m}}^{k}=C_{\text{m}}\frac{dv^{k}}{dt}+I_{\text{ion}}^{k}, \end{array}$$

where *v* is the membrane potential, *C*_m_ is the cell membrane capacitance and *I*_ion_ is the ionic current density out of the cell. Furthermore, assuming ohmic resistance along the length of the neuron, we have

(2) $$\begin{array}{r} \Delta xI_{k\;+\;1/2}=\sigma_{\text{i}}\left(u_{\text{i}}^{k}-u_{\text{i}}^{k+1}\right), \end{array}$$

where $u_{\text{i}}^{k}$ is the intracellular potential in compartment *k*, σ_i_ is the intracellular conductivity, and *I*_*k* + 1/2_ is the intracellular current density from compartment *k* to compartment *k* + 1. Applying Kirchhoff's current law, the sum of the currents flowing out of a compartment must equal the sum of currents flowing into a compartment, i.e.,

(3) $$\begin{array}{r} |\Gamma^{k}|I_{\text{m}}^{k}=l_{y}l_{z}\left(I_{k-1/2}-I_{k\;+\;1/2}\right), \end{array}$$

where |Γ^*k*^| is the membrane area associated with Ω_i,*k*_. Therefore,

(4) $$\begin{array}{r} |\Gamma^{k}|\left(C_{\text{m}}\frac{dv^{k}}{dt}+I_{\text{ion}}^{k}\right)=\frac{\sigma_{\text{i}}l_{y}l_{z}}{\Delta x}\left(u_{\text{i}}^{k\;-\;1}-2u_{\text{i}}^{k}+u_{\text{i}}^{k\;+\;1}\right). \end{array}$$

To simplify notations, we assume that *l*_*y*_ = *l*_*z*_ : = *h*, and we have

(5) $$\begin{array}{r} C_{\text{m}}\frac{dv^{k}}{dt}+I_{\text{ion}}^{k}=\frac{\sigma_{\text{i}}}{4}\frac{h}{\Delta x^{2}}\left(u_{\text{i}}^{k-1}-2u_{\text{i}}^{k}+u_{\text{i}}^{k\;+\;1}\right). \end{array}$$

Certainly, in the limit of small compartments (Δ*x* → 0), we have

(6) $$\begin{array}{r} C_{\text{m}}\frac{\partial v}{\partial t}+I_{\text{ion}}=\eta \frac{\partial^{2}u_{\text{i}}}{\partial x^{2}}, \end{array}$$

where we have introduced the conductance

(7) $$\begin{array}{r} \eta =\frac{h\sigma_{\text{i}}}{4}. \end{array}$$

The membrane potential is defined as

(8) $$\begin{array}{r} v=u_{\text{i}}-u_{\text{e}}, \end{array}$$

where *u*_e_ denotes the extracellular potential. Therefore, we can replace the intracellular potential *u*_i_ in Equation (6) by *v* + *u*_e_ to get

(9) $$\begin{array}{r} C_{\text{m}}\frac{\partial v}{\partial t}+I_{\text{ion}}=\eta \left(\frac{\partial^{2}v}{\partial x^{2}}+\frac{\partial^{2}u_{\text{e}}}{\partial x^{2}}\right). \end{array}$$

At this point a major assumption is introduced; it is assumed that the extracellular potential varies so little that it can be taken to be a constant (see e.g., Sterratt et al., 2011)^1^;

(10) $$\begin{array}{r} u_{\text{e}}\approx \text{const.} \end{array}$$

Building on this assumption we arrive at the classical Cable equation

(11) $$\begin{array}{r} C_{\text{m}}\frac{\partial v}{\partial t}+I_{\text{ion}}=\eta \frac{\partial^{2}v}{\partial x^{2}}. \end{array}$$

Note that the term we ignored in the derivation of the Cable equation is

(12) $$\begin{array}{r} I_{\text{eph}}=\eta \frac{\partial^{2}u_{\text{e}}}{\partial x^{2}}, \end{array}$$

which is referred to as the *ephaptic* current density (Holt and Koch, 1999). In the computations below we will compute this current using the EMI model and use it to quantify the effect of the assumption underlying the classical Cable equation.

#### 2.1.2. Computing the transmembrane current based on the solution of the cable equation

Next we address the problem of computing the transmembrane current based on the solution of the Cable equation. Suppose that the Cable equation is solved numerically using an implicit finite difference scheme of the form

(13) $$\begin{array}{r} C_{\text{m}}\frac{v_{n,k}-v_{n\;-\;1,k}}{\Delta t}+I_{\text{ion},n,k}=\eta \frac{v_{n,k\;-\;1}-2v_{n,k}+v_{n,k\;+\;1}}{\Delta x^{2}}, \end{array}$$

where, as above, Δ*x* denotes the spatial discretization in form of compartments, Δ*t* denotes the time-step, and *n* is used to enumerate the time steps. Then, the associated transmembrane current density is given by

(14) $$\begin{array}{r} I_{\text{m}}^{k,n}=\eta \frac{v_{n,k\;-\;1}-2v_{n,k}+v_{n,k\;+\;1}}{\Delta x^{2}}. \end{array}$$

All the methods discussed below for computing the extracellular potential rely on an approximation of this current density, but the methods differ in how the current is approximated and in the assumptions made on the extracellular domain.

#### 2.1.3. Computing the extracellular potential in terms of solving a boundary value problem; the CBV method

**Figure 2:**
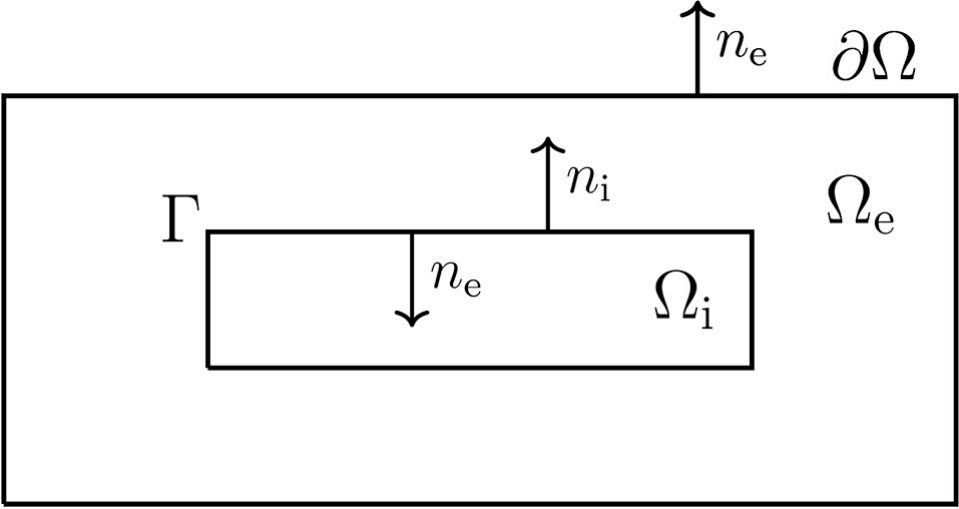
**Sketch of a simplified neuron geometry and its surroundings; the extracellular domain Ω_e_, the cell membrane Γ, and the intracellular domain Ω_i_**. The normal vector pointing out of Ω_i_, is denoted by *n*_i_ and, similarly, *n*_e_ denotes the normal vector pointing out of Ω_e_.

Consider the simplified 2D geometry illustrated in Figure 2. Our aim is now to compute the extracellular potential in Ω_e_ for the given transmembrane currents computed as explained above. The problem we have to solve is given by

(15) $$\begin{array}{r} \nabla^{2}u_{\text{e}}=0,in\Omega_{\text{e}}, \end{array}$$

(16) $$\begin{array}{r} \sigma_{\text{e}}\frac{\partial u_{\text{e}}}{\partial n_{\text{e}}}=I_{\text{m}},at\Gamma , \end{array}$$

where *I*_m_ is computed by Equation (14) and *n*_e_ is the outward pointing normal vector of Ω_e_. The boundary condition at the outer boundary of Ω_e_ will be described for the simulations presented below.

In our computations, the Laplace Equation (15) together with the boundary condition (16) is solved numerically using straightforward finite difference approximations leading to a linear system of algebraic equations. The finite difference stencil used for Equation (15) will be described below.

We will refer to this method for computing the extracellular potential as the CBV-method since it comprises the solution of the Cable equation (C) and the solution of a boundary value (BV) problem.

#### 2.1.4. Computing the extracellular potential by solving the poisson equation; the CP method

In the CBV method the transmembrane currents setting up the extracellular potential are positioned at the interface between the intracellular and extracellular domains. In the standard method for computing extracellular potentials (referred to as the CS method below), the transmembrane currents are instead assumed to be positioned at the points (or lines) at the center of the intracellular domain (Holt and Koch, 1999). One step in this direction is to replace the boundary value problem (15, 16) of the CBV method with a Poisson equation of the form

(17) $$\begin{array}{r} \nabla \cdot \left(\sigma \nabla u\right)=-C,in\Omega , \end{array}$$

where Ω = Ω_e_ ∪ Ω_i_. Here σ equals σ_i_ and σ_e_ in Ω_i_ and Ω_e_, respectively, and *u* equals *u*_i_ and *u*_e_ in Ω_i_ and Ω_e_, respectively. The problem now is how to define the current source density *C*. In order to define *C*, we start by recalling that integration by parts gives

(18) $$\begin{array}{r} \int_{\Omega }\nabla \cdot \left(\sigma \nabla u\right)\phi dV=\int_{\Gamma }\sigma \frac{\partial u}{\partial n}\phi ds-\int_{\Omega }\nabla \phi \cdot \left(\sigma \nabla u\right)dV \end{array}$$

for any smooth functions *u* and ϕ, see e.g., Grossmann et al. (2007, p. 140). By choosing ϕ = 1, and using this identity, it follows from Equation (17) that the integral of *C* must be given by

(19) $$\begin{array}{r} \int_{\Omega_{\text{i}}}CdV=-\int_{\Omega_{\text{i}}}\nabla \cdot \left(\sigma_{\text{i}}\nabla u_{\text{i}}\right)dV=-\int_{\Gamma }\sigma_{\text{i}}\frac{\partial u_{\text{i}}}{\partial n_{\text{i}}}ds=\int_{\Gamma }I_{\text{m}}ds, \end{array}$$

where *n*_i_ is the outward pointing normal vector of Ω_i_. We now want to define the source term *C* such that the identity (Equation 19) holds. To this end, we define the constants^2^

(20) $$\begin{array}{r} C_{k}=\frac{\left|\Gamma_{k}\right|}{\left|\Omega_{\text{i},k}\right|}I_{\text{m},k}, \end{array}$$

for every compartment Ω_i,*k*_, where

(21) $$\begin{array}{r} I_{\text{m},k}=\frac{1}{\left|\Gamma_{k}\right|}\int_{\Gamma_{k}}I_{\text{m}}ds \end{array}$$

is the average transmembrane current density of the compartment. Based on these constants, we can define the source term

(22) $$\begin{array}{r} C=C_{k}\text{for}x\in \Omega_{\text{i},k}. \end{array}$$

With this definition of the source term, we have

$$\int_{\Omega_{\text{i}}}Cdx=\int_{\Gamma }I_{\text{m}}ds$$

and therefore Equation (19) holds provided that the current source density *C* is defined by (22).

We can now approximate the solution of the boundary value problem (15,16) defined on Ω_e_ with the Poisson problem (17) defined on the entire Ω = Ω_e_ ∪ Ω_i_. It remains to be seen that the current flowing into the extracellular domain Ω_e_ defined by boundary condition (16) is the same as the amount of current flowing out of the intracellular domain Ω_i_ in the solution of the Poisson Equation (17). This holds, since by the definition of *C* we have

(23) $$\begin{array}{r} \int_{\Gamma }\sigma_{\text{e}}\frac{\partial u_{\text{e}}}{\partial n_{\text{e}}}ds=\int_{\Gamma }I_{\text{m}}ds=-\int_{\Gamma }\sigma_{\text{i}}\frac{\partial u_{\text{i}}}{\partial n_{\text{i}}}ds. \end{array}$$

Note that this method effectively assumes the transmembrane current to be homogeneously distributed in the intracellular domain in the computation of the extracellular potential. Again, the numerical solution of Equation (22) is obtained by the finite difference method where the right-hand side of the equation is evaluated in the mesh points. This leads to a linear system of algebraic equations.

The method of computing the extracellular potential by solving the Cable Equation (11), using the result to define the source term *C* by Equation (22), and then solving the Poisson Equation (17), will be referred to as the CP method (C is for Cable and P is for Poisson).

#### 2.1.5. Computing the extracellular potential by the point source method; the CS method

The final method for computing the extracellular potential based on the solution of the Cable equation we will consider is the point source method. This method relies on two basic assumptions; first it is assumed that all the current can be gathered in the center of each compartment; and second, it is assumed that the extracellular space is infinite. Under these assumptions, the Poisson equation can be solved analytically, see e.g., Holt (1998), Holt and Koch (1999), Gold et al. (2006), and Einevoll et al. (2013). This dramatically increases computational efficiency and thus this approach is extremely popular and completely dominates computations of extracellular potentials around neurons. Again, our aim is to assess the accuracy of this method.

By using the notation introduced above, we define current sources for each compartment by

(24) $$\begin{array}{r} c_{k}=|\Omega_{\text{i},k}|C_{k}, \end{array}$$

and define the associated Poisson problems

(25) $$\begin{array}{r} \sigma_{\text{e}}\nabla^{2}u_{\text{e},k}=-c_{k}\delta \left(r-r_{k}\right), \end{array}$$

where *r* = (*x, y, z*) and *r*_*k*_ is the center of the *k*−th compartment. The solution of this problem reads

(26) $$\begin{array}{r} u_{\text{e},k}=\frac{c_{k}}{4\pi \sigma_{\text{e}}|r-r_{k}|}, \end{array}$$

and therefore, by linearity, the extracellular potential is given by

(27) $$\begin{array}{r} u_{\text{e}}=\underset{k}{\sum }u_{\text{e},k}=\frac{1}{4\pi \sigma_{\text{e}}}\underset{k}{\sum }\frac{c_{k}}{\left|r-r_{k}\right|}. \end{array}$$

Note that |*r* − *r*_*k*_| denotes the Euclidean distance for *r* to the point *r*_*k*_. In the computations below we will refer to this method of computing the extracellular potential as the CS-method (where C is for Cable and S is for sum).

### 2.2. The extracellular-membrane-intracellular (EMI) model

The dynamics of a neuron and its extracellular surroundings can be accurately modeled by explicitly considering the Extracellular space (Ω_e_), the Membrane (Γ) and the Intracellular domain (Ω_i_); as mentioned above we call this the EMI model. Analytical examples of solutions are given by Krassowska and Neu (1994), finite element formulations are provided by Ying and Henriquez (2007), Henríquez et al. (2013), and Agudelo-Toro and Neef (2013); see also Agudelo-Toro (2012) for a detailed derivation of the model.

The main elements of the model are sketched in Figure 2. Note that Ω = Ω_i_ ∪ Ω_e_ contains a single cell, where Ω_i_ is the intracellular domain of the cell and Ω_e_ is the extracellular space surrounding the cell. We let *u*_i_ and *u*_e_ denote the intra- and extracellular potentials, and at the interface between the intracellular and extracellular domains, given by Γ, we define the membrane potential by *v* = *u*_i_ − *u*_e_. Then the electrical potential defined in Ω = Ω_i_ ∪ Ω_e_ is governed by the system

(28) $$\begin{array}{r} \nabla \cdot \sigma_{\text{i}}\nabla u_{\text{i}}=0,in\Omega_{\text{i}}, \end{array}$$

(29) $$\begin{array}{r} \nabla \cdot \sigma_{\text{e}}\nabla u_{\text{e}}=0,in\Omega_{\text{e}}, \end{array}$$

(30) $$\begin{array}{r} u_{\text{e}}=0,at\partial \Omega_{\text{e}}, \end{array}$$

(31) $$\begin{array}{r} n_{\text{e}}\cdot \sigma_{\text{e}}\nabla u_{\text{e}}=-n_{\text{i}}\cdot \sigma_{\text{i}}\nabla u_{\text{i}},at\Gamma , \end{array}$$

(32) $$\begin{array}{r} u_{\text{i}}-u_{\text{e}}=v,at\Gamma , \end{array}$$

(33) $$\begin{array}{r} I_{\text{m}}=-n_{\text{i}}\cdot \sigma_{\text{i}}\nabla u_{\text{i}},at\Gamma , \end{array}$$

(34) $$\begin{array}{r} \frac{\partial v}{\partial t}=\frac{1}{C_{\text{m}}}\left(I_{\text{m}}-I_{\text{ion}}\right),at\Gamma , \end{array}$$

where σ_i_ and σ_e_ are intra- and extracellular conductivities, *n*_i_ and *n*_e_ are the normal vectors of Ω_i_ and Ω_e_, *C*_m_ is the cell membrane capacitance, and the ion current density is given by *I*_ion_.

#### 2.2.1. Numerical methods

**Figure 3:**
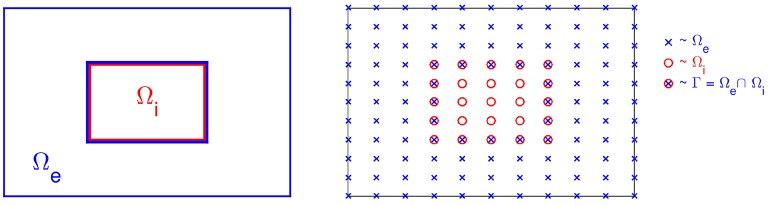
**Sketch of the computational mesh for Ω_e_ and Ω_i_; the nodes of Ω_e_ are marked by “×,” the nodes of Ω_i_ are marked by “°,” and the membrane is defined as the intersection of Ω_e_ and Ω_i_ marked by “⊗**.”

We describe the finite difference scheme for solving the system (28)–(34) in the case of passive ion currents; i.e., for a case where *I*_ion_ is linear. In this case the problem (28)–(34) is linear and it is straightforward to define a fully implicit finite difference scheme. In the description of the solution method, we will consider the 2D case illustrated in Figure 3. The extension to 3D is notationally messy but conceptually straightforward.

The system (28)–(34) can be triggered in several different ways. Since we want to compare results using the Cable equation and the EMI model, we will apply an initial condition that can be used in an identical manner for both methods. This is achieved by assuming that the membrane potential is given at time *t* = 0, and by adding a one dimensional synaptic input current.

We let $\left(u_{\text{e}}^{n,j,k},v^{n,j,k},u_{\text{i}}^{n,j,k}\right)$ denote finite difference approximations of (*u*_e_, *v, u*_i_) at (*t*_*n*_, *x*_*j*_, *y*_*k*_) = (*n*Δ*t*, *j*Δ*x*, *k*Δ*y*) for given mesh parameters Δ*t*, Δ*x* and Δ*y*. The computational nodes used for the discrete version of the system are shown in the right panel of Figure 3; nodes of the extracellular domain are marked by “×,” the intracellular nodes are marked by “◦,” and the nodes on the membrane are marked by “⊗.”

Suppose that *v* = *v*^*n*−1^ is known at time *t* = *t*_*n*−1_. The update from *t*_*n*−1_ to *t*_*n*_ is computed by solving a coupled linear system defined by a finite difference version of the system (28)–(34). In each node of the extracellular domain the elliptic Equation (29) is replaced by a finite difference scheme of the form

(35) $$\begin{array}{rc} \frac{\sigma_{\text{e}}^{j\;+\;1/2,k}\left(u_{\text{e}}^{n,j+1,k}-u_{\text{e}}^{n,j,k}\right)-\sigma_{\text{e}}^{j\;-\;1/2,k}\left(u_{\text{e}}^{n,j,k}-u_{\text{e}}^{n,j-1,k}\right)}{\Delta x^{2}}\\ + & \frac{\sigma_{\text{e}}^{j,k\;+\;1/2}\left(u_{\text{e}}^{n,j,k\;+\;1}-u_{\text{e}}^{n,j,k}\right)-\sigma_{\text{e}}^{j,k\;-\;1/2}\left(u_{\text{e}}^{n,j,k}-u_{\text{e}}^{n,j,k\;-\;1}\right)}{\Delta y^{2}}=0, \end{array}$$

where $\sigma_{\text{e}}^{j+1/2,k}=\sigma_{\text{e}}\left(\left(j+1/2\right)\Delta x,k\Delta y\right).$ Likewise, the elliptic equation (28) is replaced by a finite difference scheme of similar form (*u*_e_ replaced by *u*_i_ and σ_e_ replaced by σ_i_). The numerical scheme given by Equation (35) provides one equation for all nodes in the domain Ω_e_\Γ and (as explained above) for all nodes in the domain Ω_i_\Γ.

It remains to specify three equations for all nodes on the membrane Γ since there are three unknowns, (*u*_e_, *v, u*_i_), in each of the membrane nodes. One equation is clearly given by Equation (32); i.e., $u_{\text{i}}^{n,j,k}-u_{\text{e}}^{n,j,k}=v^{n,j,k}$ for all nodes (*x*_*j*_, *y*_*k*_) on the membrane Γ. The second equation is provided by replacing the flux-equality Equation (31) by a finite difference equation, and the third equation is the discrete version of Equation (34) in terms of an implicit scheme;

(36) $$v^{n,j,k}-\frac{\Delta t}{C_{\text{m}}}(I_{\text{m}}^{n,j,k}-I_{\text{ion}}^{n,j,k})=v^{n\;-\;1,j,k}.$$

Here, *I*_m_ is defined as a discrete version of Equation (33). Furthermore, in the passive case, the function *I*_ion_ is linear with respect to *v* and therefore the entire system is linear.

The four corners of the membrane mesh need special attention. In these nodes we define two extracellular and two intracellular flux terms; one term from the normal derivative in the *x*-direction and one from the normal derivative in the *y*-direction. Furthermore, we let the sum of the two intracellular fluxes equal the sum of the two extracellular fluxes in the flux-equality Equation (31) and let $I_{\text{m}}^{n,j,k}$ in Equation (36) be the mean of the two intracellular fluxes. In the 3D extension we similarly define three extracellular and three intracellular flux terms for the corner nodes where three membrane planes intersect, and two extracellular and two intracellular flux terms for the edge nodes where two membrane planes intersect.

In the case of simple, rectangular geometries, this numerical strategy is straightforward. However, for more complex geometries, finite element or finite volume methods should be used.

## 3. Results

In this section we will report results using the methods described above. We will start the section by investigating the error in the membrane potential introduced by ignoring the ephaptic current Equation (12).

Secondly, we will compare the extracellular potential computed by the CBV, CP, and CS methods with the solution of the EMI model. Clearly, there are a set of different assumptions underlying these methods: The CS method is unique in assuming the extracellular domain to be infinite. In order to be able to compare the results of the CS method with the other methods, we have used large extracellular domains. In order to estimate how large the domain must be, we have systematically increased the size of the extracellular space until convergence of the EMI solutions and then used the largest domain for our comparisons.

For the CS method the transmembrane currents are gathered in the center of each compartment thus giving rise to the classical formula of the solution, whereas for the CP method the transmembrane currents are distributed over each compartment, and numerical methods are used to compute the solution of the associated Poisson equation. In contrast, in the CBV and EMI methods the transmembrane currents setting up the extracellular potentials are placed at the interface between the intracellular and extracellular domains. The CBV and EMI methods are thus defined on the same domain, and the only difference lies in the proper self-consistent modeling of ephaptic effects in the EMI method.

For convenience, the abbreviations (CBV, CP, CS, and EMI) and references to the methods are summarized in Table 1.

**Table 1:** **Definition of the methods used to compute the extracellular potential**.

**Abbreviation**	**Explanation**	**Method**
CBV	Cable equation, Boundary Value problem	(11), (15), (16)
CP	Cable equation, Poisson equation	(11), (17)
CS	Cable equation, solution given by a Sum	(11), (27)
EMI	Extracellular Membrane Intracellular	(28)–(34)

### 3.1. Model parameters

We consider the Cable equation and the EMI model using the parameters given in Table 2 (unless otherwise stated). The domain Ω = Ω_i_ ∪ Ω_e_ is defined as

(37) $$\begin{array}{r} \Omega =\left[0,L_{x}\right]\times \left[0,L_{y}\right]\times \left[0,L_{z}\right], \end{array}$$

and the intracellular domain, Ω_i_, is shaped as a rectangular cuboid of size *l*_*x*_ × *l*_*y*_ × *l*_*z*_ located in the center of Ω. The ionic current density *I*_ion_ is defined as

(38) $$\begin{array}{r} I_{\text{ion}}=I_{\text{leak}}+I_{\text{syn}}, \end{array}$$

where *I*_leak_ is the leak current density given by

(39) $$\begin{array}{r} I_{\text{leak}}=g_{L}\left(v-v_{\text{rest}}\right), \end{array}$$

and *I*_syn_ is the conductance-based synaptic current density with single-exponential dynamics (see Gerstner et al., 2014) given by

(40) $$\begin{array}{r} I_{\text{syn}}=g_{s}\left(x\right)e^{-\frac{t\;-\;t_{0}}{\alpha }}\left(v-v_{\text{eq}}\right). \end{array}$$

**Table 2:** **Parameters used in the computations of the Cable equation and the EMI model**.

**Parameter**	**Value**	**Parameter**	**Value**
*L*_*x*_	60 μm	*g*_*L*_	6·10^−7^ μS/μm^2^
*L*_*y*_	20 μm	*g*_syn_	1.25·10^−3^ μS/μm^2^
*L*_*z*_	20 μm	*v*_rest_	−90 mV
*l*_*x*_	50 μm	*v*_eq_	0 mV
*h, l*_*y*_, *l*_*z*_	6 μm	*t*_0_	0 ms
Δ*x*, Δ*y*, Δ*z*	0.5 μm	α	2 ms
Δ*t*	0.02 ms	σ_i_	0.7 μS/μm
*C*_m_	2·10^−5^ nF/μm^2^	σ_e_	0.3 μS/μm

For the first 10% of the cell in the *x*-direction, *g*_*s*_(*x*) is given by the value *g*_syn_ in Table 2. On the remaining part of the membrane *g*_*s*_(*x*) is set to zero.

We use the initial condition *v* = *v*_rest_ = −90 mV for the membrane potential. In addition, we apply the boundary condition $\frac{\partial v}{\partial x}=0$ at the start and the end of the cell in the Cable equation and the boundary condition *u*_e_ = 0 on the outer boundary of Ω_e_ in the EMI, CBV and CP methods unless otherwise stated.

### 3.2. Numerical assessment of the error in membrane potential introduced by ignoring the ephaptic current

**Figure 4:**
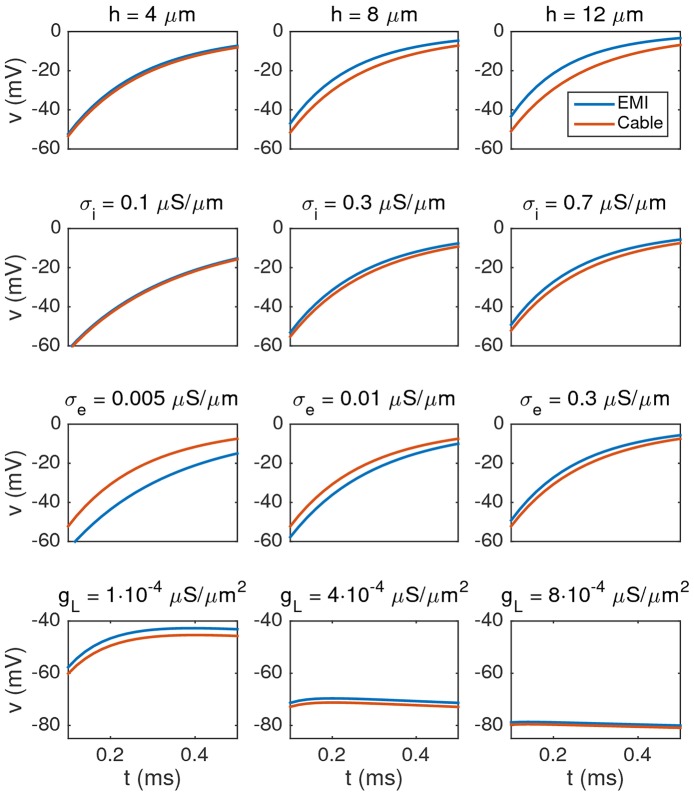
**Comparison of the membrane potential computed by solving the Cable equation (red) and the EMI model (blue) for some different values of *h*, σ_i_, σ_e_, and *g*_*L*_, where we recall that *h* = *l*_*y*_ = *l*_*z*_ (the width of the neuron)**. The plots show how the membrane potential in the compartment 25 μm from the start of the cell changes with time from *t* = 0.1 to 0.5 ms. The parameters used in the computations are given in Table 2 except for the values given above each plot. We observe that the difference between the two solutions increases when the value of *h* or σ_i_ is increased, and the difference decreases when the value of σ_e_ or *g*_*L*_ is increased. Note that in order to observe any effect of changing the value of *g*_*L*_, we increase the default value by a factor of order 100–1,000 in the lower panel of the figure.

In Figure 4 we show the membrane potential computed by solving the Cable equation and the EMI model for different values of *h*, σ_i_, σ_e_, and *g*_*L*_. The solutions are compared in the compartment 25 μm from the start of the cell (i.e., in the center of the cell in the *x*-direction). The difference is several millivolts, but it is reduced as the intracellular conductivity σ_i_ is reduced or the size *h* (recall that *h* = *l*_*y*_ = *l*_*z*_) of the neuron is reduced. Furthermore, we observe that the difference is reduced as the extracellular conductivity, σ_e_, or *g*_*L*_ is increased. These observations are consistent with our theoretical finding in an Appendix in Supplementary Material given below where we show that, under reasonable assumptions, the error introduced in the transmembrane potential by removing the ephaptic current goes like

(41) $$\begin{array}{r} O\left(\frac{h\sigma_{\text{i}}}{g_{L}\sigma_{\text{e}}}\right). \end{array}$$

To summarize, the error increases when *h* or σ_i_ are increased, and the error decreases if *g*_*L*_ or σ_e_ are increased.

### 3.3. The magnitude of the ephaptic current decreases as the extracellular conductivity is increased

As mentioned above, the derivation of the Cable equation relies on the assumption that the extracellular potential is constant, and under that assumption, the ephaptic current defined by Equation (12) can be ignored. This can also be understood on biophysical grounds as a high extracellular conductivity implies a low extracellular resistance so that potential drops due to extracellular currents driven through the extracellular medium will be small. In the limit of very large extracellular conductivities these potential drops will become negligible, i.e., the assumption of constant extracellular potentials in the standard Cable equation will become fulfilled.

In Table 3 the maximum magnitude (absolute value) of the ephaptic current (computed by solving the EMI model) is given as a function of the extracellular conductivity σ_e_, and we note that the magnitude decreases as σ_e_ is increased. In addition, we report the value of the maximum ephaptic current multiplied by the value of σ_e_ and observe that this value is close to a constant, so we have

(42) $$\begin{array}{r} I_{\text{eph}}\sim O\left(1/\sigma_{\text{e}}\right). \end{array}$$

**Table 3:** **Maximum absolute values of *I*_eph_ from time *t* = 0.02 to 1 ms as a function of σ_e_ as computed by the EMI method**.

**σ_e_ (μS/μm)**	**$I_{\text{eph}}^{\text{max}}$ (nA/μm^2^)**	**$\sigma_{\text{e}}\cdot I_{\text{eph}}^{\text{max}}$ (nAμS/μm^3^)**
0.1	0.616	0.0616
0.3	0.208	0.0623
0.6	0.104	0.0625
1.5	0.042	0.0626
3.0	0.021	0.0627

*We observe that $\sigma_{\text{e}}\cdot I_{\text{eph}}^{\text{max}}$ is close to constant for the different values of σ_e_. The parameters used in the computations are given in Table 2*.

Therefore, for very large values of σ_e_, the ephaptic current can be ignored, but the reported values of σ_e_ are in general not so large that this assumption can be generally trusted.

**Figure 5:**
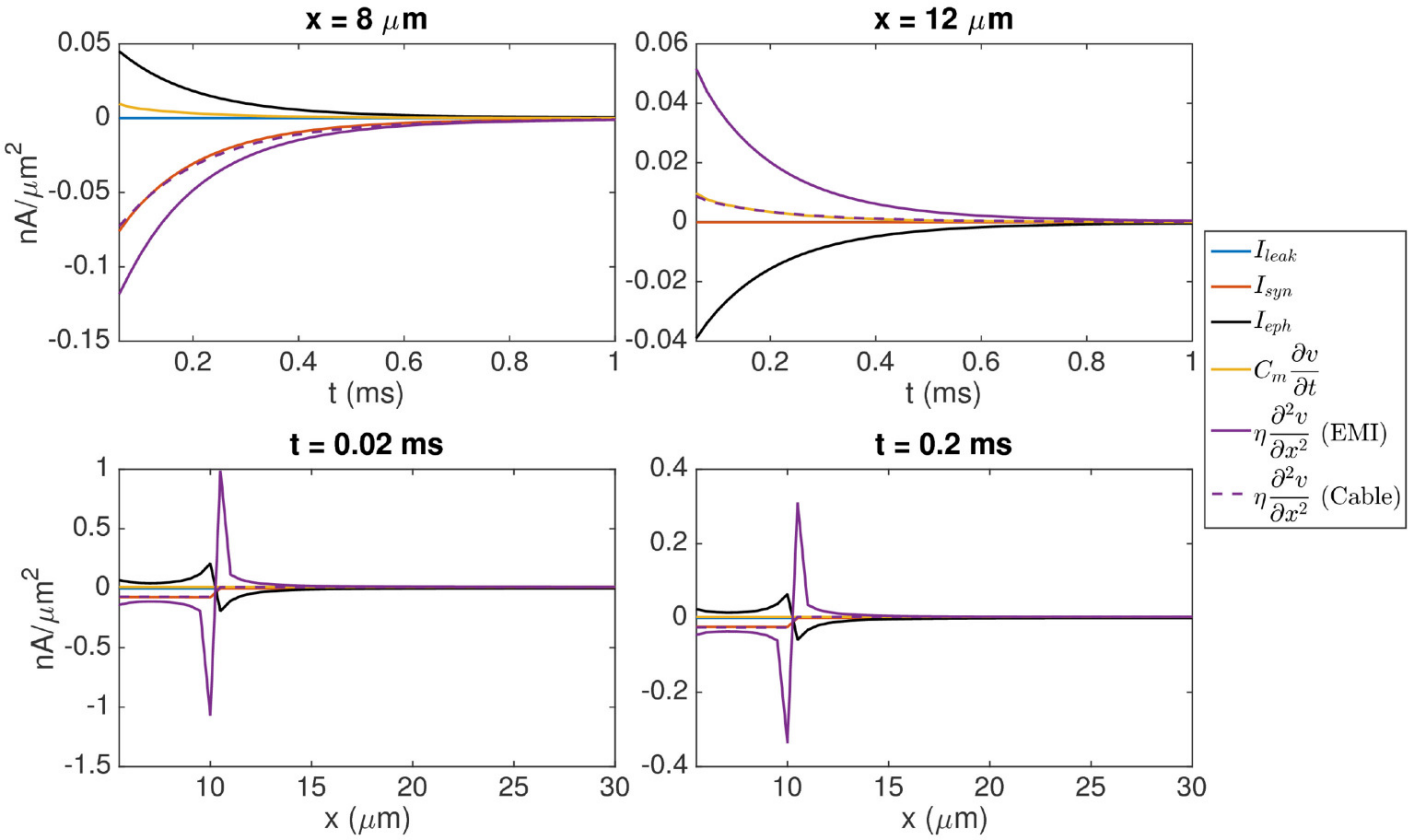
**Values of each of the terms in Equation (9)**. In the **(Upper panel)**, we show the time evolution of the terms in the point (8, 10, 7 μm) inside the synaptic input zone and the point (12, 10, 7 μm) outside the synaptic input zone. In the **(Lower panel)**, we show the values of the terms for *y* = 10 μm, *z* = 7 μm, and *x* ∈ [5 μ m, 30μm] at time *t* = 0.02 ms (left) and *t* = 0.2 ms (right). The solution of the EMI model is used to compute each of the terms. In addition, we show $\eta \frac{\partial^{2}v}{\partial x^{2}}$ for the corresponding solution of the Cable equation, where *I*_eph_ is assumed to be zero. We observe that the size of *I*_eph_ is comparable to the size of the other terms in Equation (9) and that neglecting *I*_eph_ leads to a considerable difference in the value of the term $\eta \frac{\partial^{2}v}{\partial x^{2}}$. The parameters used in the computations are given in Table 2.

It is also interesting to compare the size of the ephaptic current with the size of the other currents involved in the dynamics of the model neuron. Figure 5 shows the time evolution of each of the terms in Equation (9) and we observe that the size of the ephaptic current is comparable to the size of the other terms in the equation. The peak of the ephaptic current is located at the jump in the synaptic input.

### 3.4. Comparing the extracellular potential computed by the CBV, CP, CS, and EMI methods

In this section we will compare the extracellular potentials (EPs) computed by the EMI, CBV, CP and CS methods described above (see Table 1 for definitions of the abbreviations). When comparing the predicted extracellular potentials for the various methods, observed differences will expectedly have different model origins. For the EMI and CBV methods the key physical difference is in the lack of inclusion of ephaptic effects in the CBV method. Compared to EMI and CBV where the transmembrane currents setting up the EP are at the true membrane interface between the intracellular and extracellular domains, the CP and CS methods assume that the EP-generating currents are defined as the right-hand side of the Poisson Equation (17). For the CS method the current source density is gathered in a single point in the center of the neuronal compartment, whereas for the CP method the current density is evenly distributed over the entire compartment (See Figure 1).

#### 3.4.1. Convergence under mesh refinements

**Figure 6:**
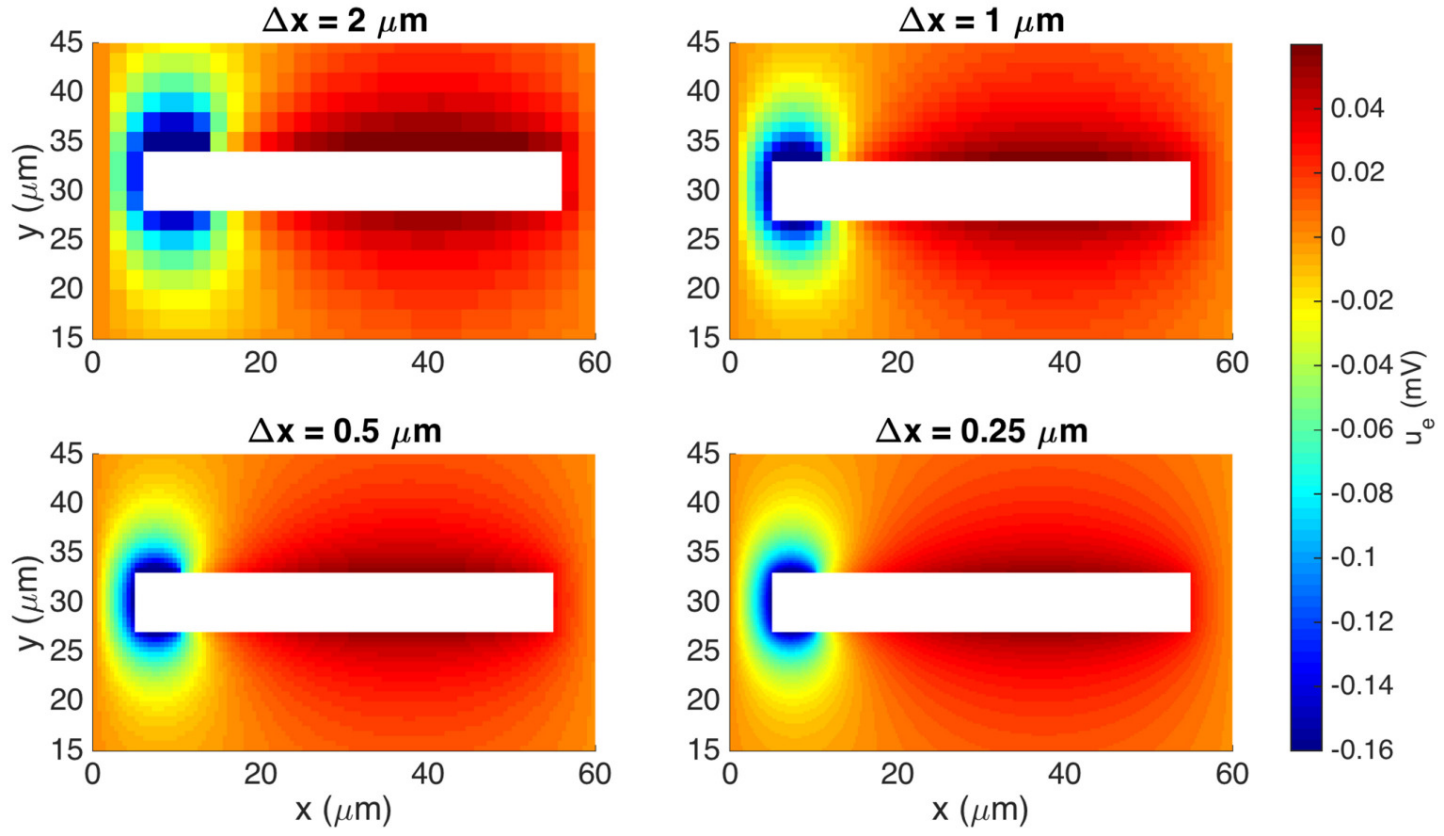
**Extracellular potential computed by the stationary EMI model for four different values of Δ*x* = Δ*y* = Δ*z***. We show the solution in a rectangle of size 60 × 30 μm on the plane in the center of the domain in the *z*-direction. The white area represents the cell. We use the parameters given in Table 2 except for an increased value of $g_{L}=3\cdot 10^{-5}$ μS/μm^2^ and a domain of size 60 × 60 × 60 μm.

In Figure 6 we show the extracellular potential computed by the EMI method for four different values of the discretization parameter Δ*x* = Δ*y* = Δ*z*. The solutions for the 0.5 μm resolution and the 0.25 μm resolution appear to be similar and we use a spatial discretization of Δ*x* = Δ*y* = Δ*z* = 0.5 μm for the rest of our computations.

To reduce the computational cost in this case, we consider the stationary version of the model, i.e., we set the time derivative in Equation (34) to zero. We use the parameter values given in Table 2, except for an increased value of $g_{L}=3\cdot 10^{-5}$ μS/μm^2^ and a domain of size 60 × 60 × 60 μm. We again let *g*_*s*_(*x*) be *g*_syn_ for the first 10% of the cell in the *x*-direction and zero elsewhere and apply the boundary condition *u*_e_ = 0 on the outer boundary of the extracellular domain.

#### 3.4.2. Convergence of the EMI solution as the domain size is increased

In the derivation of the CS method, the extracellular domain is assumed to be infinite (see Section 2.1.5). When comparing CS and EMI results, we therefore wish to compare the solution of the CS method to the solution of the EMI model as the size of the extracellular domain approaches infinity.

We again consider the stationary version of the model with the parameter values given in Table 2, except for an increased value of $g_{L}=3\cdot 10^{-5}$ μS/μm^2^ and an increased domain size.

**Figure 7:**
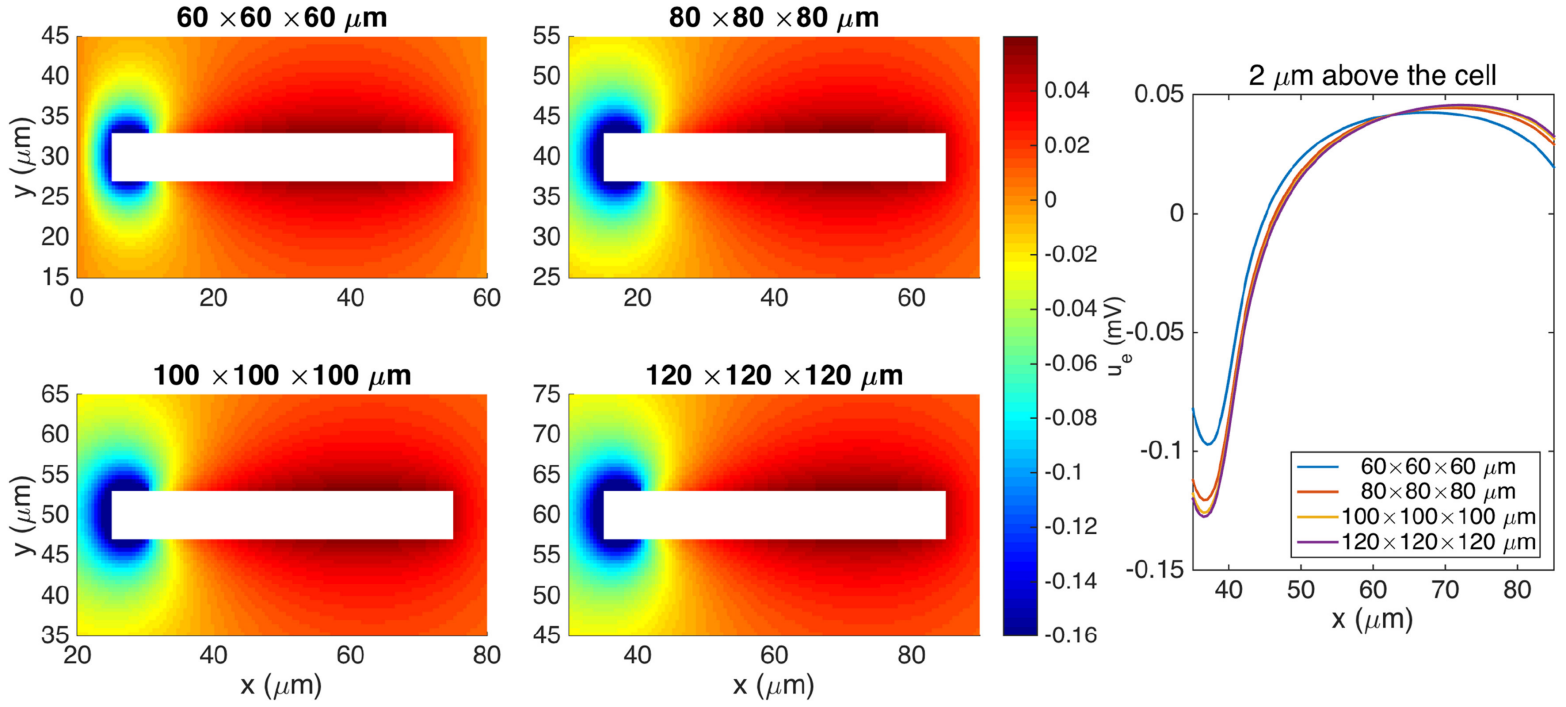
**Comparison of the extracellular potential around a neuron computed by the stationary EMI model for four different sizes of the extracellular domain**. The plots to the left show the solution in a rectangle of size 60 × 30 μm on the plane in the center of the domain in the *z*-direction. The white area represents the neuron. The plot to the right shows the extracellular potential along a line 2 μm above the neuron in the *y*-direction and in the center of the domain in the *z*-direction. The parameters used in the computations are given in Table 2 except for *L*_*x*_, *L*_*y*_, and *L*_*z*_, which are specified for each simulation, and *g*_*L*_, which is set to 3 · 10^−5^ μS/μm^2^.

Figure 7 shows the stationary solution of the EMI model for four different sizes of the extracellular domain. We observe that as the size of the extracellular domain increases, the solution of the EMI model appears to converge, and we assume that the solution for a domain of size 120 × 120 × 120μm is sufficiently large to represent the EMI solution of an infinite domain.

#### 3.4.3. One single simplified neuron

**Figure 8:**
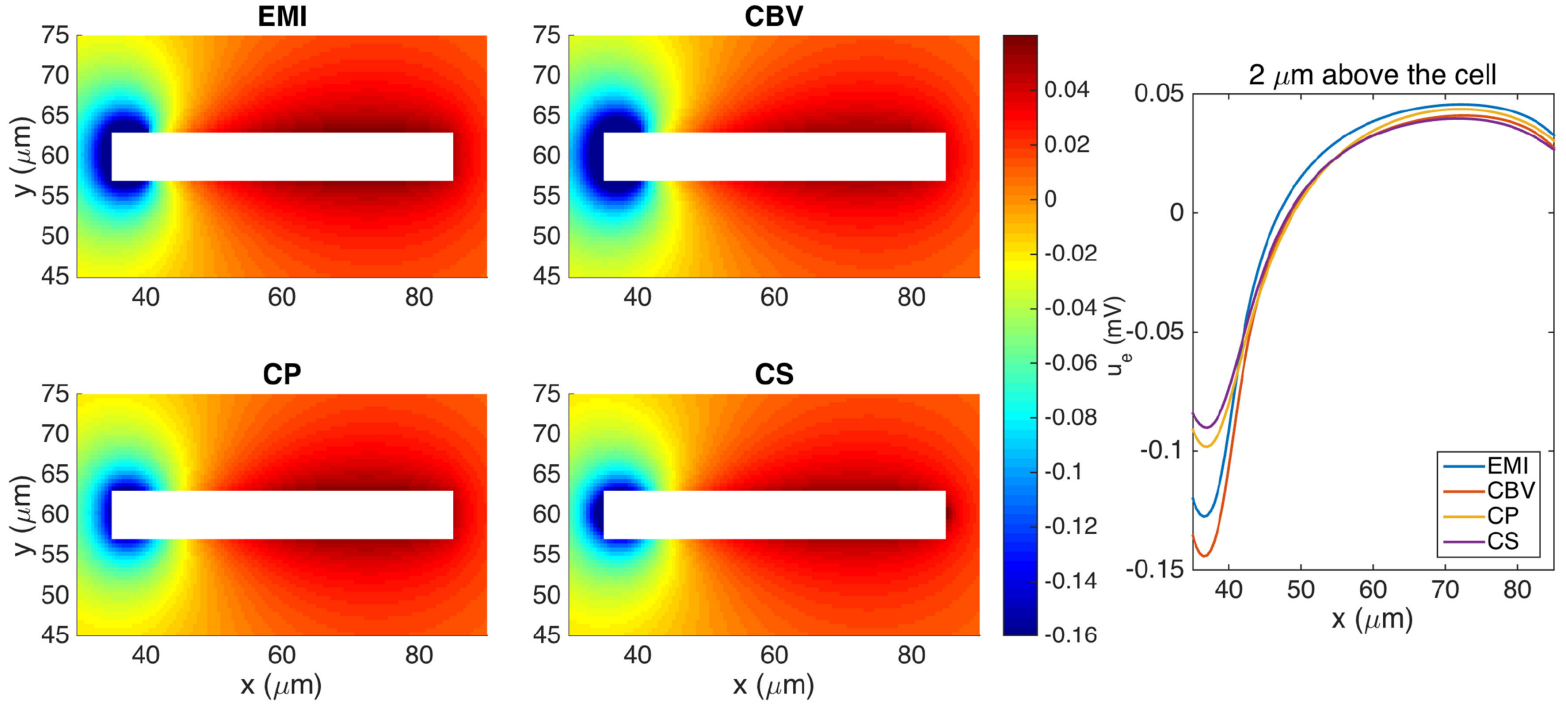
**The extracellular potential around a neuron shaped as a rectangular cuboid computed by the stationary versions of the EMI, CBV, CP, and CS methods**. The plots to the left show the solution in a rectangle of size 60 × 30 μm on the plane in the center of the domain in the *z*-direction. The white area represents the neuron. The plot to the right shows the extracellular potential along a line 2 μm above the neuron in the *y*-direction and in the center of the domain in the *z*-direction. We use the parameters specified in Table 2 except for *L*_*x*_ = *L*_*y*_ = *L*_*z*_ = 120 μm and $g_{L}=3\cdot 10^{-5}$ μS/μm^2^. The abbreviations (EMI, CBV, CP, and CS) are summarized in Table 1.

Our first test case for comparing the methods for computing the extracellular potential is a single neuron of the form given above. The extracellular potential computed by the CBV, CP, CS, and EMI methods are presented in Figure 8 (see Table 1 for definitions of the abbreviations). In Table 4 we report the maximum difference between the extracellular potential computed by the EMI model and the extracellular potential computed by each of the other methods. The deviation of the CBV result from the EMI result is smaller than the difference to the CP and CS results. Thus the largest differences appear to come from the different assumptions of placement of the transmembrane currents in the EP-generating step (compare CBV vs. CP and CS). The effect of the ephaptic current (CBV vs. EMI) is smaller.

**Table 4:** **Maximum difference between the solution for the extracellular potential in Ω_e_\Γ computed by the EMI method and each of the other methods for the test case in Figure 8**.

**Method**	**Maximum difference (mV)**	**Relative maximum difference (%)**
CBV	0.024	11.3
CP	0.058	27.7
CS	0.113	53.7

*The relative maximum differences are computed as the maximum difference divided by the maximum absolute value of the extracellular potential computed by the EMI method. The abbreviations (EMI, CBV, CP, and CS) are summarized in Table 1*.

#### 3.4.4. Two simplified neurons

**Figure 9:**
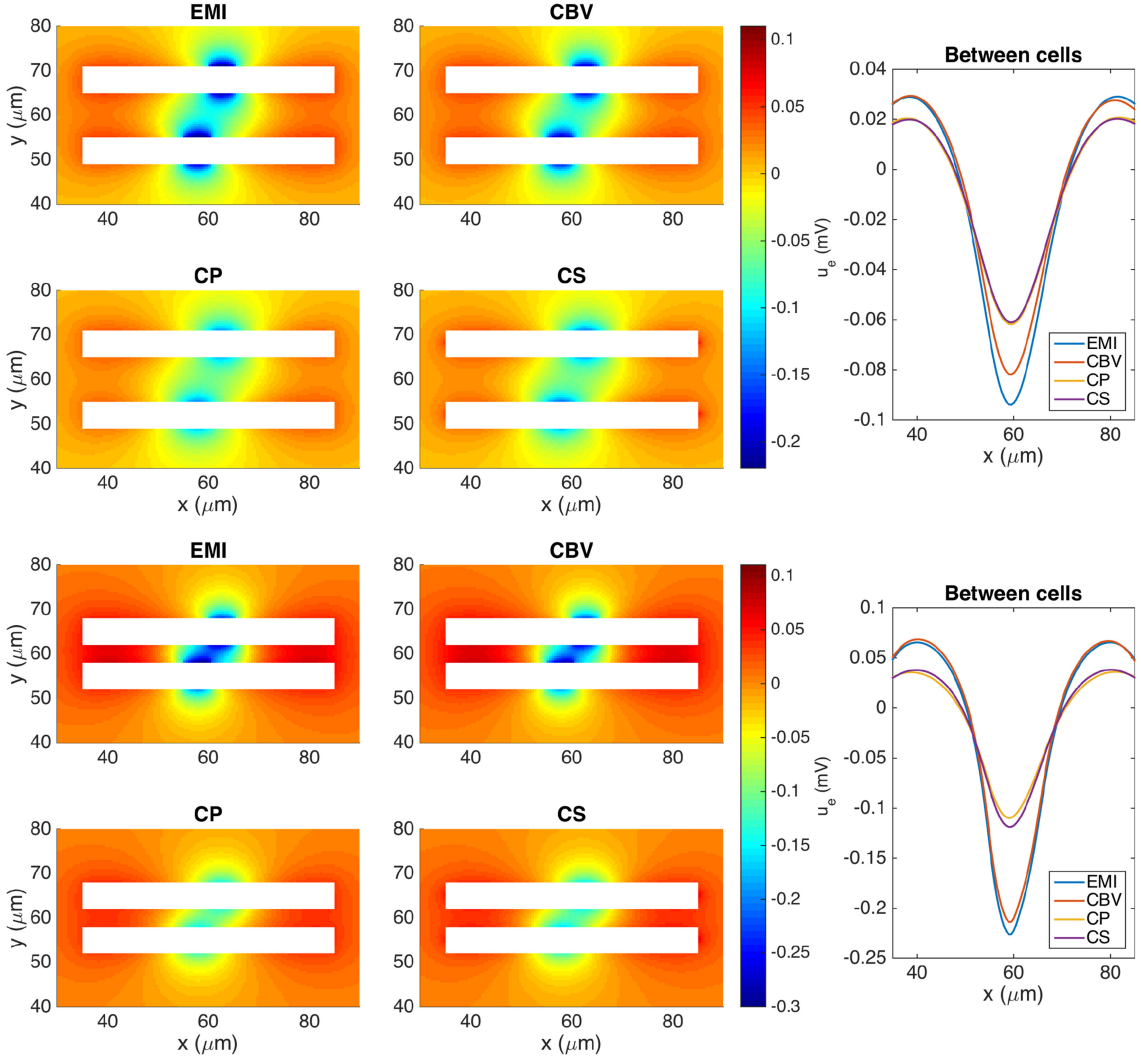
**The extracellular potential around two neurons computed by the stationary versions of the EMI, CBV, CP, and CS methods**. The plots to the left show the solution in a rectangle of size 60 × 40 μm on the plane in the center of the domain in the *z*-direction. The white areas represent the neurons. The plots to the right show the extracellular potential along the line in the center of the space between the two neurons. In the upper five plots, the neurons are separated by a distance of 10 μm in the *y*-direction, and in the lower five plots the neurons are separated by a distance of 4 μm. In all plots *g*_*s*_(*x*) is given by *g*_syn_ for *x* ∈ [55, 60 μm] and is zero on the rest of the membrane for the lower neuron. For the upper neuron *g*_*s*_(*x*) is given by *g*_syn_ for *x* ∈ [60 μm, 65 μm]. We use the parameters specified in Table 2 except for *L*_*x*_ = *L*_*y*_ = *L*_*z*_ = 120 μm and $g_{L}=3\cdot 10^{-5}$ μS/μm^2^. The abbreviations (EMI, CBV, CP, and CS) are summarized in Table 1.

In Figure 9 we show the extracellular potential around two neurons of the form given above computed by the CBV, CP, CS, and EMI methods. In the upper part of the figure the neurons are separated by a distance of 10 μ m in the *y*-direction and in the lower part the neurons are separated by a distance of 4 μm. In Table 5 we report the maximum difference between the extracellular potential computed by the EMI method and each of the other methods for the two test cases.

**Table 5:** **Maximum difference between the solution for the extracellular potential in Ω_e_\Γ computed by the EMI method and each of the other methods for the test cases in Figure 9**.

**Method**	**Maximum difference (mV)**	**Relative maximum difference (%)**
**(A) Neurons separated by 10 μm**		
CBV	0.025	12.6
CP	0.087	43.2
CS	0.086	42.4
**(B) Neurons separated by 4 μm**		
CBV	0.014	5.2
CP	0.141	52.9
CS	0.128	48.3

*The relative maximum differences are computed as the maximum difference divided by the maximum absolute value of the extracellular potential computed by the EMI method. The abbreviations (EMI, CBV, CP, and CS) are summarized in Table 1*.

As for the case with a single simplified neuron above, the deviation of the CBV result from the EMI result is seen to be smaller than the difference to the CP and CS results. Interestingly, in the lower part of Figure 9 where the distance between the two neurons is very small (4 μm), the EMI and CBV results are essentially identical in the space between the cells.

#### 3.4.5. Confined extracellular space

**Figure 10:**
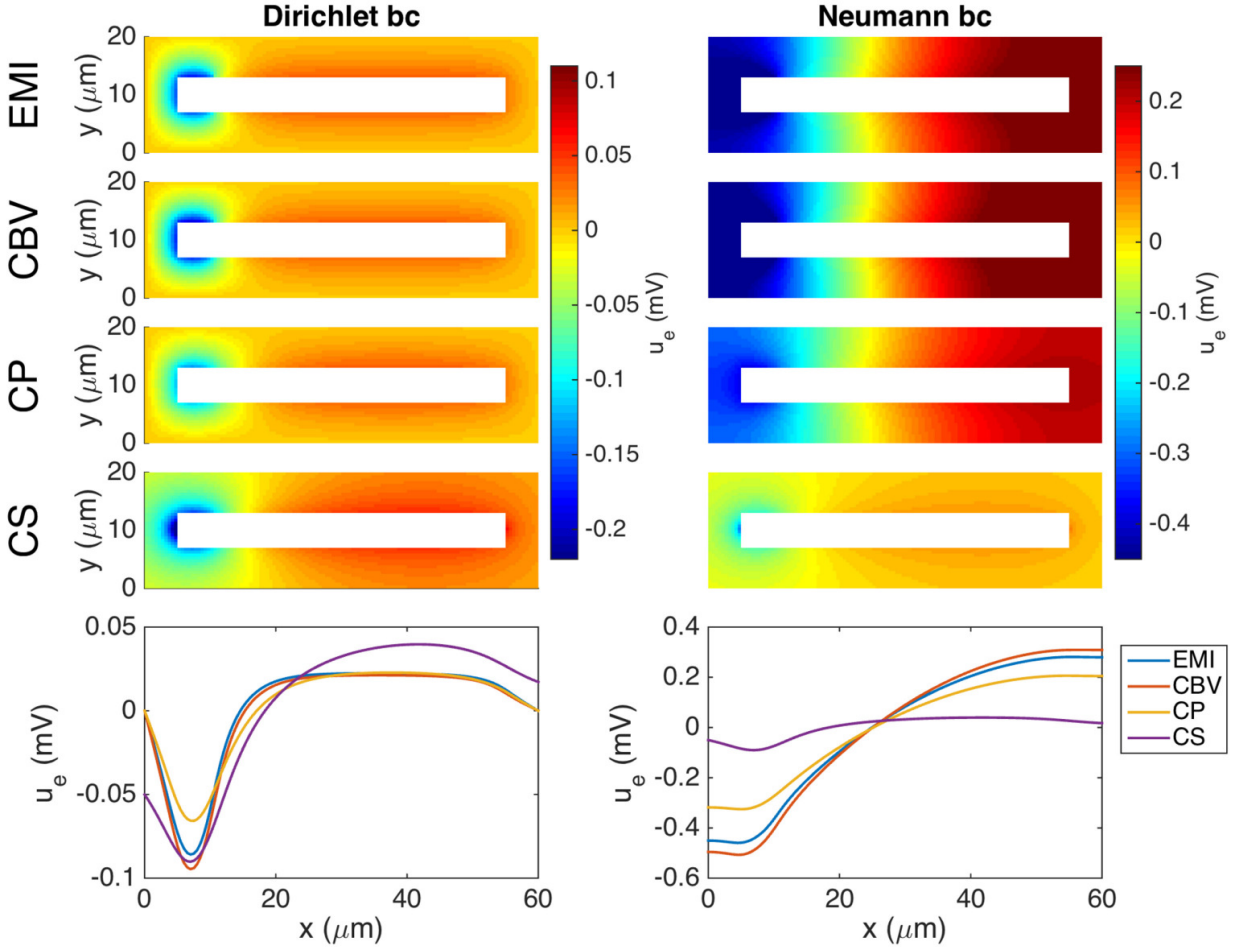
**Extracellular potential around a neuron computed by the EMI, CBV, CP, and CS methods**. We consider the stationary version of the models and the parameter values given in Table 2 except for an increased value of $g_{L}=3\cdot 10^{-5}\mu$m. A Dirichlet boundary condition, *u*_e_ = 0, is applied in the simulation in the left panel and a Neumann boundary condition, $\frac{\partial u_{\text{e}}}{\partial n_{\text{e}}}=0$, is applied in the right panel. The **(Upper panels)** show the extracellular potential in the plane in the center of the domain in the *z*-direction for each of the methods. The **(Lower panel)** shows the solution along a line 2 μm above the cell in the *y*-direction and in the center of the domain in the *z*-direction. Note that in the case of Neumann boundary conditions, we include the additional constraint $\int_{\Omega_{\text{e}}}u_{\text{e}}dV=0$ for the EMI, CBV, and CP methods in order to obtain unique solutions. The abbreviations (EMI, CBV, CP, and CS) are summarized in Table 1.

Figure 10 shows the extracellular potential around a neuron in a domain of size 60 × 20 × 20μm computed by the EMI, CBV, CP, and CS methods. The left panel shows the solution for a homogeneous Dirichlet boundary condition, and the right panel shows the solution for a homogeneous Neumann boundary condition on the outer boundary of the extracellular space.

As explained above, the CS method is founded on the assumption of an infinite extracellular space. We have therefore focused on a very large computational domain mimicking the properties of an infinite domain. Certainly, also limited domains are of interests and simulation results are given in Figure 10 using both Dirichlet and Neumann type boundary conditions. Although we present results for all four models, it is important to keep in mind that a confined domain breaks a basic assumption underlying the CS method and consequently we get very large errors, especially in the case of Neumann type boundary conditions.

Note that in the case of Neumann boundary conditions, the solution is not uniquely determined by the systems defining the EMI, CBV, and CP methods, and we expand the systems with the additional constraint

(43) $$\begin{array}{r} \int_{\Omega_{\text{e}}}u_{\text{e}}dV=0 \end{array}$$

in order to obtain unique solutions of the methods.

#### 3.4.6. Effects of the size of the synaptic input area

**Figure 11:**
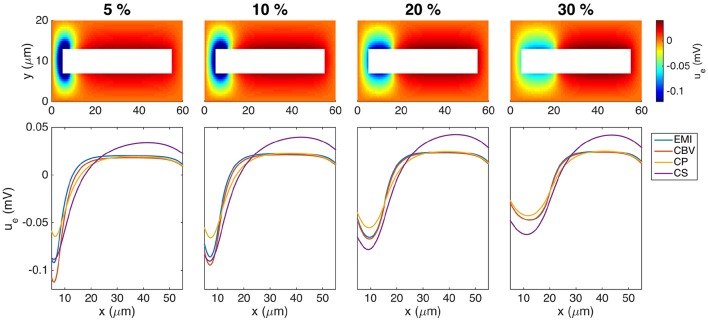
**Extracellular potential around a neuron with a synaptic input area of length 5, 10, 20, and 30% of the total cell length**. The **(Upper panel)** shows the extracellular potential computed by the EMI method in the plane in the center of the domain in the *z*-direction. The **(Lower panel)** shows the solution for each of the methods along a line 2 μm above the cell in the *y*-direction and in the center of the domain in the *z*-direction. The figure shows the solution of the stationary version of the models using the parameter values given in Table 2 except for an increased value of $g_{L}=3\cdot 10^{-5}\mu$m. We apply a homogeneous Dirichlet boundary condition on the outer boundary of the extracellular domain. The abbreviations (EMI, CBV, CP, and CS) are summarized in Table 1.

In Figure 11 we show the extracellular potential surrounding a neuron for four different sizes of the synaptic input area. The upper panel shows the extracellular potential computed by the EMI method, and the lower panel shows a comparison of the extracellular potentials computed by each of the methods along a line above the neuron. We note from the simulations that the results are qualitatively similar for all different sizes of the synaptic input region. Therefore, we choose to focus on the 10% synaptic input region as the base case for our simulations.

#### 3.4.7. Simulation time

Table 6 shows the CPU time for the simulations shown in the left panel of Figure 10 using a direct and an iterative solver.

**Table 6:** **CPU time in seconds for the EMI, CBV, CP, and CS methods**.

	**System size**	**CPU time (s) Direct solver**	**CPU time (s) Iterative solver**
EMI	208,491	34.9	15.5
CBV	101 + 191,422	29.6	1.9
CP	101 + 203,401	35.2	3.3
CS	101 + 0	1.0	1.0

*In the third column the linear systems of the EMI, CBV, and CP methods are solved using direct Gaussian elimination, and in the fourth column the linear systems are solved using the bistable conjugate gradient stabilized method with an incomplete LU preconditioner and a relative tolerance of *10*^−*5*^. The second column reports the number of unknowns in the linear systems to be solved in each of the methods. In the EMI model, we solve a coupled system for the extracellular, membrane, and intracellular potentials simultaneously. In the CBV, CP, and CS methods, on the other hand, we first find the membrane potential by solving the Cable equation consisting of 101 compartments. Then, we find the extracellular potential by solving an equation for each node in the extracellular or entire domain, for the CP and CBV methods, respectively. In the CS method, the extracellular potential is given directly from the solution of the Cable equation by an explicit formula. The parameters used in the simulation are given in Table 2 except for an increased value of $g_{L}=\text{3}\cdot {\text{10}}^{-\text{5}}\mu$m. The table reports the solution time for the stationary versions of the models with homogeneous Dirichlet boundary conditions*.

The EMI model is clearly much more computationally expensive than the classical CS method. This is expected because the EMI model involves solving a large coupled system of equations, whereas the CS method only requires solving the Cable equation which involves a much smaller number of unknowns. After solving the Cable equation, the CS methods assumes that the extracellular potential may be found directly by the explicit formula (27), so no further equations has to be solved.

Moreover, in the computations reported in the table, the extracellular potential is computed for all nodes in the mesh. In the CS method this is not necessary, and the CPU time for the CS method could possibly be further reduced by only computing the values for the points of interest. This is not possible for the EMI method (or the CBV or CP methods) because the systems of equations has to be solved for all nodes in order to find the solution in a single point.

In contrast, the simulation time for the CBV and CP methods are more comparable to that of the EMI model, at least for the direct solver. This is because these methods also rely on solving a linear system of equations for all nodes in the extracellular domain or the entire domain for the CVB and CP methods, respectively.

The extra complexity introduced in the EMI model by solving for the membrane, intracellular and extracellular potentials simultaneously is apparent, however, when an iterative method is applied to solve the linear system. The fourth column of Table 6 shows the solution time for each of the methods using the bistable conjugate gradient stabilized method with an incomplete LU preconditioner. In this case, the CBV and CP methods are much faster than the EMI method.

## 4. Discussion

In the present paper we have compared four different methods for computing neural dynamics. In the numerically comprehensive EMI model the intracellular and extracellular dynamics are solved self-consistently and the membrane potentials and extracellular potential are computed simultaneously. For the other methods (CBV, CP, CS; see Table 1 for definitions of abbreviations) the membrane potential is first computed using the Cable equation, and the resulting transmembrane currents are used in a second step to compute the extracellular potential.

In the CBV method the transmembrane currents are placed on the interface between the intracellular and extracellular domains, and the only difference with the EMI model is the lack of self-consistency in the two-step computational scheme inherent in the CBV scheme, that is, the transmembrane currents are first computed using the Cable equation assuming a constant extracellular potential, while a non-constant potential (both in space and time) is computed in the second step.

For the CP and CS methods an additional assumption is made in the second step, namely that the effect of the transmembrane currents are assumed to be represented in terms of currents source densities. Specifically, for the CP method, the current source density is distributed evenly over a neuronal compartment and a numerical scheme is used to solve the resulting Poisson Equation (17), and for the CS method the source density is concentrated in a single point and thus the classical sum formula (27) of the solution can be applied.

### 4.1. Ignoring the ephaptic current

#### 4.1.1. Error in membrane potential introduced by ignoring the ephaptic current

To study the error introduced by ignoring the ephaptic current in the Cable equation, we compared the membrane potential computed by solving the Cable equation to the corresponding solution of the EMI model. In our simple test case, we found that the membrane potential computed by the Cable equation could differ several millivolts from the solution of the EMI model and that the magnitude of the error seems to decrease with the value of the intracellular conductivity, σ_i_, and the cell width, *h*. This suggests that the Cable equation is applicable for computing the membrane potential for sufficiently thin dendrites.

#### 4.1.2. Ephaptic current decreases with increasing extracellular conductivity

In the derivation of the Cable equation, it is assumed that the extracellular conductivity σ_e_ is so large that the extracellular potential varies very little in space and can be assumed to be a constant. As a result, the ephaptic current *I*_eph_ will be zero and may be removed from the model. In our numerical simulations of the EMI model, we confirmed that the size of *I*_eph_ decreases when the value of σ_e_ is increased (see Table 3). In fact, we found that the maximum absolute value of *I*_eph_ appeared to be inversely proportional to the value of σ_e_. However, we also observed that the magnitude of *I*_eph_ was similar in size to the other currents involved in the model (see Figure 5). This suggests that *I*_eph_ is not negligible for the stylized neuron geometries and model parameters chosen here.

### 4.2. Error in neglecting ephaptic currents

The CBV and EMI methods are defined on identical domains, and the key physical difference between the methods is the absence of ephaptic effects in the CBV method. Comparisons of computed extracellular potentials indeed show such ephaptic effects of varying magnitudes, both for the extracellular potential outside a single activated neuron (Figure 8) and between two activated neurons (Figure 9).

### 4.3. Effects of position of transmembrane currents

To explore the effects of assumed positions of transmembrane currents, it is easiest to compare results from the three methods, i.e., CBV, CP, and CS, where the transmembrane currents in all cases are found from the Cable equation. Here effects from the ephaptic current are in all cases absent. For the present examples we observe that CS and CP results are typically quite similar, but both quite different from the CBV results (Figures 8, 9). From the point-source formula in (27) we see that the contribution to the extracellular potential from a point current source is inversely proportional to distance, and it is thus not surprising that this difference in assumed source positions has a sizeable effect on the predicted extracellular potentials.

### 4.4. Effects of size of extracellular domain

Both in the CP and CBV methods (as well as in the EMI model) the extracellular domain is finite, while in the CS method the extracellular domain is infinite so that the solution of the Poisson equation can be given as an explicit sum. With a very small extracellular domain, corresponding to a small piece of brain tissue embedded in an insulator, large deviations from the infinite-domain results will be observed (Figure 7; see also Figure 10).

In order to compare results with the CS method, we here computed the EMI solution for gradually larger domains until the solutions appeared to converge. Further, we regarded the converged solution as the solution of the EMI problem for an infinite extracellular space, i.e., we estimated that the difference of the results for the largest considered domain and a (hypothetical) infinite domain was negligible for the present purposes. Roughly speaking, convergence was obtained for an extracellular space extending twice the length of the cable in every direction.

### 4.5. The simplified geometry

Today, simulations of neurons typically use much more complex and realistic geometries than what has been applied here. Already in Clark and Plonsey (1968) were able to analytically evaluate the extracellular potential of a cylindrical neuron, and it is certainly of interest to evaluate the models and methods discussed here in more realistic geometries. The generic limitation of the finite difference method used here is that it is hard to apply, correctly, to non-rectangular geometries. In Agudelo-Toro and Neef (2013), the finite element method is used and this gives much more freedom to represent realistic geometries. However, the code used in Agudelo-Toro and Neef (2013) required extremely fine times steps and we therefore focused on simplified geometries where the problem could be solved using a reasonable number of time steps. The solution of the EMI model using an implicit formulation will likely reduce the time step restrictions and this is subject for ongoing investigations.

Another limitation in the present study is the size of the extracellular space. This space is actually quite limited (see e.g., Syková and Nicholson, 2008), but the assumption of an infinite extracellular space is necessary for the application of the classical CS method (summation method), and thus we have used very large extracellular domains in order to provide fair comparisons with the classical model at the cost of simulating more realistic volumes.

### 4.6. Neural tissue

The EMI model provides a useful framework for accurate computations of the electrophysiology of a small number of cells and their surroundings. It is, however, very hard to apply this methodology to neural tissue consisting of huge numbers of cells. In simulations of cardiac tissue, the Bidomain approach has successfully been applied to simulate the electrophysiology, see e.g., Keener and Sneyd (2009), Franzone et al. (2014), Sundnes et al. (2006); Roth (2013), and Trayanova (2011). Recently, a similar approach has been applied to neural tissue, see Meffin et al. (2014) and Tahayori et al. (2014). Most likely, some form of homogenization process is needed to derive tractable mathematical models for neural tissue.

### 4.7. Possible additive effects for non-linear membrane dynamics

We have focused on a linear membrane model in order to highlight the effect of removing the ephaptic current in the simplest possible case. More generally, the question is whether *ephaptic coupling would constitute a “feedback” mechanism with electric fields altering the activity of the same neural elements that gave rise to them in the first place*, see Anastassiou and Koch (2015). For a linear model, this feedback mechanism was recently found to be the *small but not negligible*, see Goldwyn and Rinzel (2016), which clearly is consistent with our findings. However, the effect may very well be larger for non-linear models of the membrane dynamics; small electric fields can be amplified by non-linear effects, see Radman et al. (2007). At present, we have not conducted systematic simulations using a non-linear membrane model.

### 4.8. Other assumptions

We note also that the current study was limited to standard simulation frameworks in neuroscience, where intra- and extracellular currents are assumed to be purely Ohmic, so that Equations (28) and (29) apply in the bulk solutions. That is, we did not include possible contributions from advective currents, displacement currents and ionic diffusion currents. These currents are typically neglected, as they are believed to play negligible roles for the system electrodynamics under most biophysically relevant conditions. However, computational studies have indicated that at least ionic diffusion could, in some scenarios, influence electrical potentials (see e.g., Qian and Sejnowski, 1989; Bédard and Destexhe, 2013; Halnes et al., 2013, 2016; Pods et al., 2013; Pods, 2017). These effects were not accounted for in the current study. We also confined our simulations to linear, passive membranes even if it known that active voltage-gated channels affect the extracellular potential; see e.g., Ness et al. (2016).

## 5. Conclusion

We have compared various methods for computing membrane potentials and extracellular potentials. For the simple test cases considered here, non-negligible errors were observed when neglecting ephaptic effects, i.e., when comparing results from the EMI model with the CBV model building on results from the Cable equation. Further, substantial differences in the predicted extracellular potentials were observed depending on whether transmembrane current sources were assumed to be placed in the center of the neural compartment or at the membrane interfaces. This study motivates further analysis of the errors for computations based on more realistic representations of the geometry and dynamics of the neurons using the EMI model.

## Author contributions

Writing paper: AT, KJ, GH, GE. Programming: KJ, LP, GL. Simulation: KJ, AT. Numerical methods: AT, KJ, JS, GL. Discussion: AT, KJ, TM, AE, GH, GE. Outline of research: all authors.

### Conflict of interest statement

The authors declare that the research was conducted in the absence of any commercial or financial relationships that could be construed as a potential conflict of interest.

## Footnotes

^1^Assuming that the extracellular potential is a linear function of position would result in the same simplified model.

^2^Note that *C*_*k*_ is a constant defined on the compartment Ω_i,*k*_; it is constant in space but varies in time.
