# Supplementary material for: An Evaluation of the Accuracy of Classical Models for Computing the Membrane Potential and Extracellular Potential for Neurons
Source: Front Comput Neurosci. 2017 Apr 24;11:27. doi: 10.3389/fncom.2017.00027 (PMC5401906; doi:10.3389/fncom.2017.00027)
Supplement: Supplementary file 1 [file DataSheet1.pdf]

Appendix: Theoretical considerations concerning the effect of removing the ephaptic current

In this appendix, we will provide theoretical arguments indicating the asymptotic nature of the errors introduced by removing the ephaptic current. These arguments are founded on strong assumptions on the analytical properties of the solutions and rigorous mathematical arguments would require *a priori* proofs of these properties. We therefore emphasize that the arguments provided here just indicate relations and it is an open problem to rigorously prove these relations mathematically.

As discussed above, the key step in deriving the classical Cable model is to remove the ephaptic current, I_{eph}. We have given computational evidence indicating that

$$I_{\text{eph}} \sim O\left(1/\sigma_{\text{e}}\right).$$

This relation can also be derived from the classical summation formula (see Equation (27) in the paper). If we assume that (27) holds and assume that $\frac{\partial^2 u_{\text{e}}}{\partial x^2}$ is uniformly bounded, we find that

$$I_{\text{eph}} = \eta \frac{\partial^2 u_{\text{e}}}{\partial x^2} = O\left(\frac{h\sigma_{\text{i}}}{\sigma_{\text{e}}}\right),$$

where we have used that $\eta = \frac{h\sigma_{\text{i}}}{4}$; recall that $h = l_y = l_z$ (the width of the neuron).

Next, our aim in this appendix is to provide a rough estimate of the error introduced in the membrane potential by removing the ephaptic current given by (12) in the paper. The theoretical bound will be based on the assumption that the term $\frac{\partial^2 u_{\text{e}}}{\partial x^2}$ in (12) is bounded independently of the parameter η.

In order to derive the bound, we compare the two models given by

$$C_{\text{m}} v_t + I_{\text{ion}}(v, x, t) = \eta \left(v_{xx} + u^e_{xx}\right), \tag{1}$$

and

$$C_{\text{m}} \bar{v}_t + I_{\text{ion}}(\bar{v}, x, t) = \eta \bar{v}_{xx}. \tag{2}$$

Here subscript t represents the derivative with respect to t and subscript xx represents the double derivative with respect to x. For simplicity, we assume that both models are equipped with the boundary condition $v = v_{\text{rest}}$ at $x = 0$ and $x = l_x$.

By subtracting (1) from (2), we find that the error

$$e = \bar{v} - v$$

is governed by

$$C_{\text{m}} e_t + g(x,t)e = \eta e_{xx} - \eta u^e_{xx}, \tag{3}$$

with the boundary condition $e(0,t) = e(l_x,t) = 0$, initial condition $e(\cdot,0) = 0$, and where

$$g(x,t) = g_L + g_s(x)e^{-\frac{t-t_0}{\alpha}}.$$

By multiplying (3) by e and integrating over the length of the neuron, we get

$$\frac{1}{2}C_{\text{m}}\frac{d}{dt}\int_0^{l_x} e^2 dx + \int_0^{l_x} g(x,t)e^2 dx = -\eta\int_0^{l_x} e_x^2 dx - \eta\int_0^{l_x} e u^e_{xx} dx. \tag{4}$$

First, we note that

$$\int_0^{l_x} g(x,t)e^2 dx \geqslant g_L\int_0^{l_x} e^2 dx, \tag{5}$$

and secondly, we use the Poincaré inequality (see e.g. [1]) to find that

$$\frac{l_x^2}{2}\int_0^{l_x} e_x^2 dx \geqslant \int_0^{l_x} e^2 dx. \tag{6}$$

In order to estimate the last term of (4), we note that, for any a, b and $\varepsilon \neq 0$, we have

$$0 \leqslant \left(\varepsilon a - \frac{b}{\varepsilon}\right)^2 = (\varepsilon a)^2 - 2ab + \left(\frac{b}{\varepsilon}\right)^2$$

and therefore

$$ab \leqslant \frac{1}{2}\left((\varepsilon a)^2 + \left(\frac{b}{\varepsilon}\right)^2\right).$$

By using this inequality with $a = e$, and $b = u^e_{xx}$, we find that

$$-\int_0^{l_x} e u^e_{xx} dx \leqslant \int_0^{l_x} \left|e u^e_{xx}\right| dx \leqslant \frac{1}{2}\left(\int_0^{l_x} (\varepsilon e)^2 dx + \int_0^{l_x}\left(\frac{u^e_{xx}}{\varepsilon}\right)^2 dx\right). \tag{7}$$

We define

$$E(t) = \int_0^{l_x} e^2 dx,$$

and note that, by $(4, 5, 6, 7)$, we have

$$\frac{1}{2}C_{\mathrm{m}}E' \leqslant -\frac{2}{l_x^2}\eta E - g_L E + \frac{\eta\varepsilon^2}{2}E + \frac{\eta}{2\varepsilon^2}F_0, \tag{8}$$

where we have introduced

$$F_0 = \max_t \int_0^{l_x} (u_{xx}^e)^2\, dx.$$

Again, if we assume that the extracellular potential is faithfully represented by the classical summation formula (27), we have

$$F_0 = O(1/\sigma_{\mathrm{e}}^2).$$

Equation (8) can be written as

$$\frac{1}{2}C_{\mathrm{m}}E' \leqslant (A\varepsilon^2 - B)E + C/\varepsilon^2$$

with $A = \eta/2, B = 2\eta/l_x^2 + g_L$ and $C = \eta F_0/2$. Provided that $B > A\varepsilon^2$ this ODE will be bounded by the steady state

$$E^*(\varepsilon^2) = \frac{C}{\varepsilon^2(B - A\varepsilon^2)}.$$

Choosing $\varepsilon^2 = B/2A$ in order to minize this upper bound, it follows that

$$E(t) \leqslant E^*\left(\frac{B}{2A}\right) = \frac{4AC}{B^2} = \frac{\eta^2}{(\frac{2\eta}{l_x^2} + g_L)^2}F_0.$$

Since, $F_0 = O(1/\sigma_{\mathrm{e}}^2)$, we find that for small values of η we have

$$E(t) \leqslant O\left(\frac{\eta^2}{g_L^2\sigma_{\mathrm{e}}^2}\right) = O\left(\frac{h\sigma_{\mathrm{i}}}{g_L\sigma_{\mathrm{e}}}\right)^2,$$

where we recall that $h = l_y = l_z$ represents the width of the neuron. Finally, we conclude that

$$\|e(t)\| = E^{1/2}(t) = O\left(\frac{h\sigma_{\mathrm{i}}}{g_L\sigma_{\mathrm{e}}}\right).$$

This estimate indicates that the error introduced by removing the ephaptic current is reduced as h or σ_{i} are reduced, and it is reduced if σ_{e} or g_L is increased.

References

[1] Aslak Tveito and Ragnar Winther. *Introduction to Partial Differential Equations; a Computational Approach*, volume 29. Springer-Verlag, second edition, 2009. 392 pages.
